# Working Women in Large Towns

**Published:** 1891-02-07

**Authors:** 


### Working Women in Large Towns.

XIX.—WHAT THE GENERAL PUBLIC MAY DO.

Excellent as are the Aids for Working Women mentioned in our last articles, it is evident to all who study the question that they must be regarded in the light of "samples," rather than of "goods delivered," so utterly disproportionate are they, in the present stage of their growth, to the large and rapidly-increasing class with which they are concerned. There is a pressing need, not only for more funds, but for more workers; and surely those helpers and those funds cannot fail to come, when once the public really knows the state of the case, and hears of remedies which patient and wise-minded investigators of the subject believe capable of alleviating, if not of doing away with, the present distress. But it is not our intention here to urge, except by the way, that direct help should be given, either in money or in work, to these schemes for assisting working women. Doubtless we do not see our responsibilities in this matter as we should; doubtless there are many who now stand idle in the market place, to whom the work cries aloud for help, and upon whose heads, if they will not listen, will be the blood of their neglected sisters. What we are concerned with just now, however, is the pointing out of certain ways in which the general public, however occupied with daily cares and business, may yet do something to help. And by the general public we do not mean the general public *as a whole*, upon whom of course rests the duty of making laws, and seeing that they are carried out, but rather the general public as a mass of units, each with his individual duties, not only to himself and his family, but to the community. What we want to impress upon the general public's mind is this. Various schemes for bettering the condition of down-trodden workers may be devised – have been devised —by those who have enquired into the matter; but unless they are backed up by a sensible and kindly public, they have but little chance of success.

Take, for instance, the efforts which have been made to start co-operative workrooms, where needle-women shall be paid a fair wage for a day's labour—and by a fair wage is meant, remember, not a high wage, but one sufficient to put the worker in decent comfort. What does the general public do? It weeps at the "Song of the Shirt"; it waxes indignant over the hardships of Mr. Besant's Melenda, the ruin of her unfortunate mate, Lizzie; and then—it will not trouble itself so far as to patronise the co-operative workrooms, or, if it does enter their doors, will perhaps turn over all the work and walk out again, remarking that it is "too dear." We are not inventing; the managers of such workrooms know such visitors only too well. Has it really come to this—that some among us *prefer* to live on the flesh and blood, aye, and the very souls, of their sisters? Is it too hard to label such conduct, as one writer has done,—"a refined cannibalism?" We can no longer plead ignorance; the broad facts are known to all but those who deliberately stop their ears. For very shame, one would think, if for nothing else, we must shrink from branding ourselves as inhuman traders upon the helplessness and misery of others.

With regard to the evils caused by irregular employment, we have already remarked that consumers as well as employers might do something to lessen these. Ladies are often extremely inconsiderate in giving their orders to dressmakers, milliners, etc., waiting till the last moment, so as to be sure of being in the very latest fashion, and then demanding to have their work finished "right away," as the Yankees say. Could they but realise the misery and danger of a protracted "slack season," and then the long hours of toil, and the harassed, "driven" feeling of the work-girls when at last the "busy season" sets in, they would surely take the trouble to give their orders a little earlier, even at the risk of appearing a trifle old-fashioned and "dowdyish."

As to the poor over-worked shop-assistants, it is as a rule the lower classes who do their shopping late in the evening, and who are thus responsible for the terribly long hours in this department of labour; but we do think that the upper classes also show great want of consideration for the young women who serve them, giving them many journeys where one would suffice, asking to look at various things which they never mean to buy, and altogether behaving in a thoughtless, capricious, and entirely selfish manner. If they could only understand what the life of the girl who serves them really is, surely they would look upon her in a new light, and think not only of themselves, but of her.

In conclusion, a word or two about servants. If the public is convinced, as we think it must be, that domestic service is safer and more suitable for young girls than other kinds of work, why do not heads of households try to make service more attractive? When they see that "places" are shunned, and other employments eagerly snatched at until competition brings wages down almost to starvation point, why do they not ask themselves whether they and their systems may not be to blame, instead of putting down the evils of the present state of things to the wildness and general "contrairiness" of girl-nature? No doubt they have often much to put up with from their servant-girls; but the old adage remains true, "good mistresses make good servants," and nothing can be worse for all parties concerned, than for mistress and maid to look upon each other as natural enemies. Many of the little servant-girl's faults are due rather to ignorance and incompetence than to wilful wickedness; and if only those mistresses who have time to spare would make it their business to teach the little maid her work, instead of constantly scolding her for doing it badly, they would be doing good service, not merely for themselves and their maids, but for the world in general. As we have said before, it is unskilled labour, in this as in other paths of life, which is so unsatisfactory to every one concerned. Readers of Howells' novels will remember the dulness and *ennui* which a rise in life brought to Mrs. Silas Lapham, and the strong desire which she felt to leave her grand drawing-room and return to the household duties which were now taken from her, and given over to her maids. We believe there are many women who, like her, thirst for some useful occupation, and who would find real happiness (though that should not be their primary object), in following the example of two or three good ladies known to the present writer, and transforming ignorant girls into bright, capable little servants.

We have said above that service should be made attractive. By this we by no means intend to commend the practices of certain housewives, who are so thankful to get a servant who understands her work, that they trouble themselves about nothing else, giving her "evenings out" when she likes to ask for them, and winking at anything and everything so long as it does not interfere to any serious extent with her work. On the contrary, we would urge that mistresses should feel more, instead of less, responsible for the real welfare of their servants; that a mere business relation between them—so much work on the one side, so much money on the other—is not all that is needed. The comfort, the instruction, and the amusement of the little world below stairs should be seen to by the mistress, even as are those of her own daughters. If no story-books are provided for the little servant-girl, how is she likely to resist the cheap and tempting literature which so often degrades her mind? If no amusement, no society, is put in her way, what wonder if she slips out after dark, and spends the stolen minutes with companions who will probably only lead her astray? Or what wonder, again, if she finally decides to fly that dull, dark kitchen, and goes back to her old home, joining the crowds of girls seeking employment in factories or workshops, and telling all her friends that "service is so terrible dull, they'd better never try it!"

Every mistress, again, should make it her business to find out the addresses of respectable Servants' Homes, to which she can refer girls who are leaving her, and may not have another place to go to. If she would only take the trouble to do this, many a slip might be prevented,—many a tale of misery and of shame which would appal her, in after-years, if she ever came to know it.

Other ways in which we may, as individuals, minister to the welfare of working women will no doubt occur to such of our readers as have read this series of articles with attention and sympathy. In cases of distress, two things are needed: first, knowledge of the exact circumstances; secondly, intelligent and ready sympathy on the part of those who acquire this knowledge. The former we have done our best, so far as space would allow, to supply; as for the latter, we trust that it may have been roused in many a heart, and that readers of The Hospital, as well as other members of the body politic, will do all that in them lies to find some practical outcome of it.

